# Which Front-of-Package Nutrition Label Is Better? The Influence of Front-of-Package Nutrition Label Type on Consumers’ Healthy Food Purchase Behavior

**DOI:** 10.3390/nu15102326

**Published:** 2023-05-16

**Authors:** Fen Liao, Han Li

**Affiliations:** 1 School of Insurance, Shandong University of Finance and Economics, Jinan 250014, China; 2 College of Economics and Management, Huazhong Agricultural University, Wuhan 430070, China

**Keywords:** front-of-package nutrition label, evaluative nutrition label, objective nutrition label, spokesperson type

## Abstract

To help consumers understand the healthfulness of food and make healthy food choices, manufacturers are increasingly providing front-of-package nutrition labels. However, not all types of front-of-package nutrition labels can promote consumers’ healthy food purchase behavior. We explored the impact of front-of-package nutrition label type on the consumer purchase behavior of healthy food through three experiments. The results show that evaluative (vs. objective) front-of-package nutrition labels can improve consumer purchase intention and willingness to pay for healthy food. The spokesperson type moderates the influence of front-of-package nutrition labels on consumers’ healthy food purchase behavior. Specifically, when the spokesperson type is a typical consumer, consumers are more willing to buy healthy foods with evaluative nutrition labels than those with objective nutrition labels. When the spokesperson type is a star, consumers are more willing to buy healthy food with objective nutrition labels (vs. evaluative nutrition labels). Finally, this study provides feasible suggestions for marketers to select appropriate front-of-package nutrition labels.

## 1. Introduction

The prevalence of high-calorie food such as fast food has led to a yearly rise in diet-related diseases worldwide, resulting in enormous economic losses [1]. The costs associated with being overweight or obese in the United States exceed USD 1.7 trillion [2]. Similarly, it is estimated that the indirect economic loss due to overweight or obesity will account for 8.73% of GNP in China by 2025 [3]. Therefore, more and more countries have adopted food nutrition labels, the main tool helping consumers understand the nutritional value, to increase consumers’ choice of healthy foods [4].

However, although some countries have been providing nutrition labels (such as Nutrition Facts Panel) for decades [5], not all of them are popular with consumers. Consumers usually ignore or cannot understand the detailed Nutrition Facts Panel (NFP) shown on the back of packaged foods [6]. By contrast, front-of-package (FOP) nutrition labels have gradually become an effective tool for food manufacturers to help consumers choose healthy food [7] because the FOP nutrition label is considered to require less effort and time to process [5].

FOP nutrition labels can be further divided into objective nutrition labels (the quantitative objective information about calories and other key nutrients, directly extracted from the Nutrition Facts Panel) and evaluative nutrition labels (the interpretive information about the overall healthfulness of the product or certain product attributes) according to their differences in content and format [8]. More importantly, prior studies have not reached a consistent conclusion on which FOP nutrition label leads to higher healthy food purchase behavior. Some scholars find that evaluative nutrition labels (vs. objective nutrition labels) can lead to a higher healthy food purchase intention [9]. Other scholars have come to the opposite conclusion, arguing that consumers seem more willing to buy healthy food with objective nutrition labels compared with evaluative nutrition labels [10].

Based on this gap, the current research aimed to explore the relationship between FOP nutrition labels and consumers’ healthy food purchase behavior and to build a framework to determine when an objective nutrition label is better for stimulating consumers to buy healthy food and when an evaluative nutrition label is better. Specifically, the first objective of our study was to conduct a systematic empirical examination of the effect of FOP nutrition label type (evaluative vs. objective) on consumers’ healthy food purchase intention or willingness to pay. Our second objective was to examine the moderating effects of spokesperson type.

## 2. Literature Review and Conceptual Framework

### 2.1. Literature Review

There are many kinds of FOP nutrition labels, including Health Star Rating [11], Traffic Light [12], Warning [13], Healthy Choice [14], Facts Up Front [15], Daily Intake Guide [16], and Guideline Daily Amount [17] labels. Regarding the content and format of FOP nutrition labels, they can be divided into two types: objective nutrition labels and evaluative nutrition labels [8]. An objective nutrition label is a brief, impartial, and objective nutrition label taken from the mandatory Nutrition Facts Panel, including Facts Up Front [14]; Daily Intake Guide [16]; and Guideline Daily Amount [17] labels. An evaluative nutrition label can provide consumers with an interpretation of a product’s overall healthfulness (e.g., a more or less healthy choice) or certain product attributes (e.g., low or high fat), including Health Star Rating [11]; Healthy Choice [14]; and Traffic Light [12] labels.

Multiple studies have focused on the influence of FOP nutrition label type on consumer health consumption behavior [8,9], but a unified conclusion has not been reached. Some studies suggested that evaluative (vs. objective) nutrition labels are more effective in promoting the consumer purchase behavior of healthy food. Bialkova et al. suggested that, compared with Healthy Choice labels, the Daily Guideline is more effective in increasing consumer purchase intention for healthy food [10]. Mauri found that a sugar teaspoon can indicate sugar levels more effectively, thus helping consumers make healthier choices compared with Traffic Light labels [18]. Dixon et al. showed that in contrast to the Health Star Rating, consumers tend to perceive sugar content information as more informative and persuasive, thereby reducing the consumption of sugary beverages [19]. 

However, several studies have drawn the opposite conclusion that an evaluative nutrition label is more effective in increasing the consumer choice of healthy food. Feunekes et al. showed that Healthy Choice labels may lead to consumers having a higher purchase intention for healthy food compared with the Daily Guideline [9]. Ducrot et al. revealed that, compared with Traffic Light and Nutri-Score labels, the use of the Guideline Daily Amount can cause a decrease in the overall nutrition quality of the food they purchase [20]. Hwang showed that evaluative disclosure led to significantly less favorable consumer evaluations of selected unhealthy food than did the absolute disclosure [21]. In summary, there may be significant differences between evaluative and objective nutrition labels in certain aspects, leading to changes in consumer healthy food purchase behavior.

### 2.2. Hypotheses Development

#### 2.2.1. Nutrition Label Type

According to the definition of the FOP nutrition label type, an objective nutrition label presents objective quantitative information on food calories and other key nutrients, so an objective nutrition label is informative (the degree of information richness [22]). For example, Feunekes et al. show that the nutrition information of the Guideline Daily Amount is detailed [9]. In contrast, an evaluative nutrition label provides interpretive information about the overall healthfulness of a product or a certain attribute. Evaluative nutrition information is usually offered by food manufacturers according to the standards established by the authoritative organization. Therefore, the evaluative nutrition label provides easy-to-understand nutrition information (perception of the cognitive effort needed to comprehend the information [23]). For example, it is easy for consumers to understand the nutritional value of food with health claims [24].

However, although an evaluative nutrition label provides explanatory information that is easier for consumers to understand than an objective nutrition label, evaluative nutrition information only explains a few selected nutrients or summarizes the overall healthfulness of the food [25], failing to fully and accurately represent the key nutritional components [26]. In contrast, an objective nutrition label provides more objective and detailed information, but it lacks a specific explanatory description [27]. Many researchers have found similar evidence when comparing the effects of the two types of nutrition labels on consumer behavior. For example, Neal et al. suggest that consumers perceive Health Star Ratings as easier to understand than Daily Intake Guidelines [16]. Van Herpen and van Trijp find that consumers are more likely to trust a Nutrition Table (vs. Healthy Choice or Traffic Light labels) because they think that a Nutrition Table details the nutritional value and is more instructive [28]. Therefore, compared with an evaluative nutrition label, an objective nutrition label is more informative but less easy to understand.

Meanwhile, the provision of nutrition labels can also be viewed as one of the marketing strategies of manufacturers. Studies show that consumers will make inferences about the motives of manufacturers’ behavior based on the clues presented by the manufacturers [29]. For example, Cheema and Patrick suggest that the way in which a manufacturer frames its promotions affects consumers’ perceptions of the manufacturer’s responsibility and professionalism [30]. Thus, when consumers see an FOP nutrition label, they are also likely to infer the manufacturer’s motives for providing it. Prior studies show that the use of inaccurate information will make people think that the information provider is evading responsibility or has insufficient knowledge about the information [31]. Confident individuals tend to be more optimistic and have the courage to express their views and present more information [32]. Thus, consumers may speculate that manufacturers providing richer information are more confident in their products. At the same time, people will judge the other party’s competence according to their confidence in presenting information [33], and people usually infer competence from the confident behavior of others [34,35]. Therefore, we believe that consumers will view manufacturers that provide richer information as more competent. On the other hand, manufacturers that go the extra mile to provide consumers with more understandable information (for example, calculating, aggregating, and interpreting the calorie and nutrient content of foods) and manufacturers that put more effort into informing consumers are also considered to care more about them [36,37]. Thus, we propose that consumers will infer that manufacturers providing easier-to-understand information are warmer. Overall, we posit that consumers will perceive manufacturers providing evaluative nutrition labels (vs. objective nutrition labels) as warmer and less competent.

In turn, both competence and warmth can make consumers have a positive attitude towards the brand [38,39], and thus consumers are more willing to purchase the products of the manufacturer [40]. In other words, compared with manufacturers providing objective nutrition labels, manufacturers providing evaluative nutrition labels are perceived as warmer and less competent, thus affecting consumers’ purchase intention positively and negatively, respectively. However, according to the warm priority effect [41], consumers will think that warmth is more important than competence when making interpersonal judgments about manufacturers [42]. Therefore, we believe that, compared with the provision of objective nutrition labels, the provision of evaluative nutrition labels will lead to higher purchase intention and willingness to pay. Formally, we hypothesize the following:

**Hypothesis** **1** **(H1).** *Compared with objective nutrition labels, providing evaluative nutrition labels is more likely to increase consumers’ healthy food purchase behavior.*

#### 2.2.2. Moderating Role of Spokesperson Type

According to the above inference, consumers will consider manufacturers providing objective nutrition labels as more competent and manufacturers providing evaluative nutrition labels as warmer. Therefore, this study speculates that the influence of the FOP nutrition label type on consumers’ purchase behavior also depends on consumers’ relative weighting of the competence and warmth perception of manufacturers. Thus, if consumers weigh competence more, they are more willing to purchase food from the manufacturers that provide objective nutrition labels; in contrast, if consumers focus on warmth, they may prefer food with evaluative nutrition labels.

Moreover, as an important marketing strategy of manufacturers, the spokesperson type may have an influence on the relative weight on competence and warmth in consumers’ decisions, which further moderates the influence of the FOP nutrition label type on consumers’ purchase behavior. Spokesperson types can be divided into the typical consumer and star [43]. A star spokesperson means that the spokesperson is a celebrity with certain public recognition, while a typical-consumer spokesperson means that the spokesperson is a real or potential consumer of the product. Typical-consumer spokespersons will make consumers feel more similar [44], and consumers will feel closer to people sharing similar characteristics with them [45]. Hence, when manufacturers choose the typical consumer as the spokesperson, the consumers’ perceived psychological distance to the manufacturers is closer. In contrast, the star spokesperson has high authority [46] and a certain popularity [47]. Therefore, there is a great social distance between consumers and stars [48], which leads to a higher perception of psychological distance between consumers and celebrity spokespersons. 

Furthermore, previous studies show that when consumers are at a great psychological distance from manufacturers, they attach more importance to the competence of the manufacturers [49]. When the psychological distance between consumers and manufacturers is relatively close, consumers attach more importance to the warmth of manufacturers [50]. For example, individuals favor the warmth of close friends while caring more about the competence of distant strangers. Therefore, when manufacturers choose a star as the spokesperson to promote their food, consumers pay more attention to the manufacturer’s competence, and an objective nutrition label (vs. evaluative nutrition label) is more likely to promote consumers’ healthy food purchase behavior. In contrast, when manufacturers choose a typical consumer as the spokesperson to promote their food, consumers attach more importance to the warmth of the manufacturers, and an evaluative nutrition label (vs. objective nutrition label) is more likely to promote consumers’ healthy food purchase behavior. Formally, we hypothesize the following:

**Hypothesis** **2** **(H2).** *Spokesperson type (star vs. typical consumer) moderates the influence of the nutrition label type on consumers’ healthy food purchase behavior.*

**Hypothesis** **2a** **(H2a).** *When manufacturers choose a star as the spokesperson to promote their food, consumers are more willing to purchase healthy food from the manufacturer providing an objective nutrition label rather than an evaluative nutrition label.*

**Hypothesis** **2b** **(H2b).** *When manufacturers choose a typical consumer as the spokesperson to promote their food, consumers are more willing to purchase healthy food from the manufacturer providing an evaluative nutrition label rather than an objective nutrition label.*

## 3. Methodology

Three studies were conducted to test the hypotheses in our research. In study 1, we compared the difference between evaluative and objective nutrition labels in information richness and comprehensibility. We next investigated the relatively positive effect of evaluative nutrition labels (vs. objective nutrition labels) on consumers’ healthy food purchase behavior in study 2. In study 3, we determined the moderation effect of spokesperson type.

### 3.1. Study 1: Differences between Evaluative and Objective Nutrition Labels

#### 3.1.1. Method

We conducted an experiment to verify the difference in information comprehensibility and richness between objective and evaluative nutrition labels. A total of 144 Chinese participants (52% female, Mage = 34) were recruited from Credamo platform in this study. The experiment used a single-factor (FOP nutrition label type: evaluative vs. objective) between-subjects design. 

**Figure 1:**
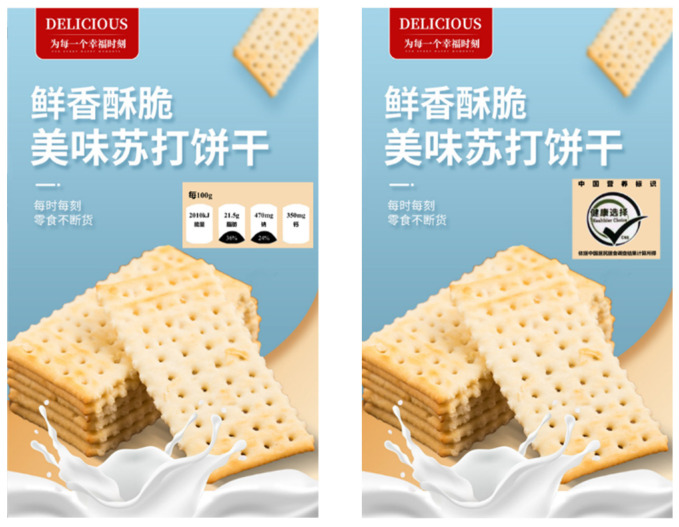
Publicity picture of soda biscuits.

First, the participants were randomly divided into two groups and asked to read a brief introduction about food manufacturers (not the definition of an FOP nutrition label). Then, participants were shown a picture with either a Healthy Choice or Facts Up Front label randomly assigned (see Figure 1). Next, each group of participants answered 2 questions (“This nutrition information is easy to understand/informative for evaluating the overall nutrition level of soda biscuits”; 1 = strongly disagree, 7 = strongly agree). Finally, demographic information was reported.

#### 3.1.2. Results

Differences between evaluative and objective nutrition labels: The results of ANOVA revealed that, compared with the Healthy Choice label (M = 3.49, SD = 1.668), participants thought that the Facts Up Front label provided more information (M = 4.17, SD = 1.639; F (1, 142) = 6.014, *p* < 0.05). Compared with the Facts Up Front label (M = 3.61, SD = 1.669), participants considered the Healthy Choice label easier to understand (M = 4.36, SD = 1.661; F (1, 142) = 7.313, *p* < 0.01).

Therefore, the results were consistent with the prediction of this experiment, providing empirical support for the differences between objective and evaluative nutrition labels proposed in our study. Namely, for consumers, an objective nutrition label is more informative, while an evaluative nutrition label is easier to understand.

#### 3.1.3. Discussion

Study 1 determined the differences in information richness and comprehensibility between objective and evaluative nutrition labels, which were similar to the findings of previous research [9,24]. Feunekes et al. demonstrate that an objective nutrition label is more informative than an evaluative nutrition label [9]. In contrast, Pang and Hammond suggest that, compared with an objective nutrition label, an evaluative nutrition label is easier to understand [24]. However, there are still differences between our findings and previous studies. We found that the difference in information richness and comprehensibility between objective and evaluative nutrition labels can coexist.

However, study 1 only demonstrated that objective and evaluative nutrition labels were fundamentally different in information richness and comprehensibility. Which type of FOP nutrition label was more likely to increase consumer willingness to buy healthy food remained unclear. Therefore, the following study 2 investigated the effect of the FOP nutrition label type on consumer purchase behavior of healthy food.

### 3.2. Study 2: The Influence of FOP Nutrition Label Type on Consumer Purchase Behavior

#### 3.2.1. Method

The main objective of study 2 was to test hypothesis 1, namely the influence of objective and evaluative nutrition labels on consumers’ healthy food purchase behavior. Specifically, we expected that the provision of evaluative nutrition labels by manufacturers would lead to higher purchase intention and willingness to pay compared with the provision of objective nutrition labels. A total of 145 Chinese participants (59% female, Mage = 36) from the Credamo platform completed this study in exchange for monetary compensation. We also used a single-factor (FOP nutrition label type: evaluative vs. objective) between-subjects design.

First, participants were asked to imagine buying soda biscuits. Participants were then randomly assigned to one of two shopping conditions. Participants were told that food manufacturer “A” had recently released a soda biscuit and were shown a publicity picture for the soda biscuit (see Figure 1). Afterwards, participants were asked to take a short survey. The survey included measures of purchase intention, willingness to pay, and relevant demographic variables. Following prior research [51], participants indicated their purchase intention on a seven-point Likert scale. We used 4 items to measure purchase intention (e.g., “I am likely to purchase these soda biscuits”; 1 = “strongly disagree” and 7 = “strongly agree”; α = 0.774). Following Netemeyer et al. [52], participants completed 1 question measuring the willingness to pay (“Assuming that the average price of soda biscuits on the market is 15 RMB/packet (400 g), I would like to buy this packet of soda biscuits at the price of _ RMB/packet (400 g)”). Finally, we expressed our gratitude to the participants.

#### 3.2.2. Results

An ANOVA with the FOP nutrition label type as the independent variable and purchase intention and willingness to pay as the dependent variables revealed that participants were more likely to buy soda biscuits with a Healthy Choice label (M = 4.26, SD = 1.070) than a Facts Up Front label (M = 3.90, SD = 1.380, F (1, 143) = 3.102, *p* < 0.1). Participants were more willing to pay more for soda biscuits with a Healthy Choice label (M = 18.36, SD = 4.863) than a Facts Up Front label (M = 16.72, SD = 5.751, F (1, 143) = 3.416, *p* < 0.1).

**Figure 2:**
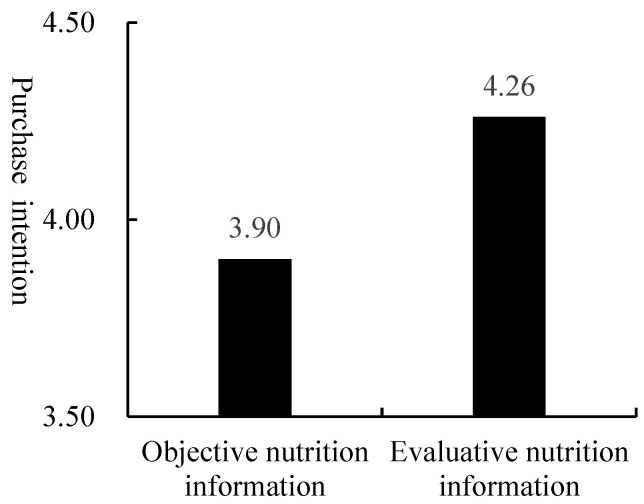
Consumers’ purchase intention for soda biscuits.

**Figure 3:**
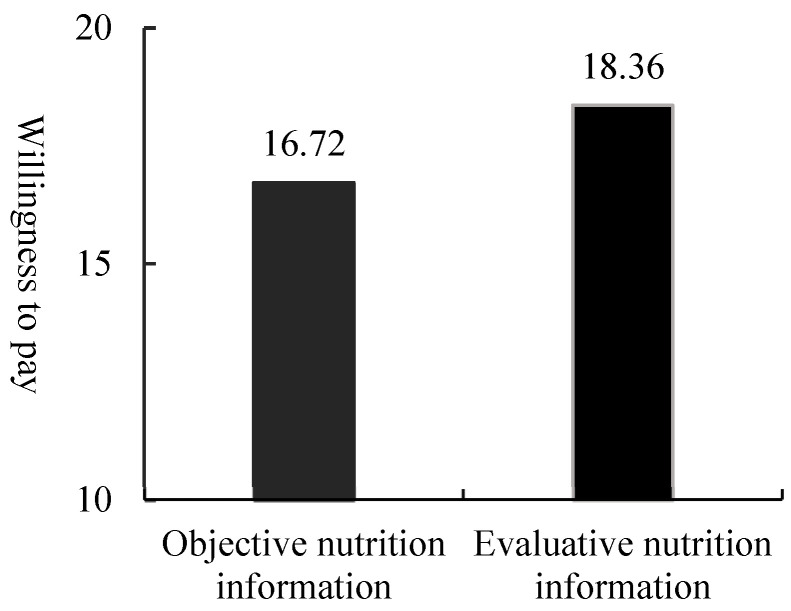
Consumers’ willingness to pay for soda biscuits.

Therefore, compared with objective nutrition labels, evaluative nutrition labels can better enhance consumers’ healthy food purchase behavior. Hence, hypothesis 1 was verified (see Figure 2 and Figure 3).

#### 3.2.3. Discussion

Based on the differences in information richness and comprehensibility between objective and evaluative nutrition labels, study 2 further verified the influence of the FOP nutrition label type on consumers’ healthy food purchasing behavior.

Although we found similar findings to parts of previous research that suggested that the provision of evaluative nutrition labels can lead to higher purchase intention or more purchases of healthy food [20,21], we thought that our findings are a trade-off between information comprehensibility and information richness. This proposition was indirectly verified by introducing the moderator spokesperson type and revealing the moderation effect in study 3.

### 3.3. Study 3: Moderating Effect of Spokesperson Type

#### 3.3.1. Method

The primary purpose of study 3 was to test hypothesis 2, namely the moderating effect of spokesperson type. We expected that when manufacturers chose a star as the spokesperson to promote their healthy food, the provision of objective nutrition labels compared with evaluative nutrition labels would lead consumers to have higher purchase intention and willingness to buy. In contrast, when manufacturers chose a typical consumer as the spokesperson to promote their healthy food, consumers would be more willing to buy the food with an evaluative nutrition label. A total of 296 Chinese participants (57% female, Mage = 36) from the Credamo platform completed this study in exchange for monetary compensation. This study utilized a 2 (FOP nutrition label type: evaluative vs. objective) × 2 (spokesperson type: star vs. typical consumer) between-subjects design.

Participants were randomly assigned to one of four conditions. First, we manipulated the FOP nutrition label type in the same way as in study 2. Next, we manipulated the advertising spokesperson type of soda biscuits through a paragraph. In the star spokesperson condition, the participants read: “Recently, manufacturer “A” chose a famous star in China as its soda biscuits spokesperson”. In the typical-consumer spokesperson condition, the participants read: “Recently, manufacturer “A” chose an ordinary soda biscuit consumer as the spokesperson”. Then, the participants completed the measurement items of purchase intention (α = 0.724) and willingness to pay (same as study 2). Finally, we measured relevant demographic variables and thanked participants.

#### 3.3.2. Results

An ANOVA on consumers’ purchase intention with the FOP nutrition label type and spokesperson type as independent variables revealed a significant main effect of the FOP nutrition label type (F (1, 292) = 11.319, *p* < 0.01). The main effect of the spokesperson type was nonsignificant (F (1, 292) = 0.370, *p* = 0.544). We observed the hypothesized FOP nutrition label type × spokesperson type interaction (F (1, 292) = 36.520, *p* < 0.01). A further simple effect analysis illustrated that participants in the star spokesperson condition were more willing to purchase soda biscuits with a Facts Up Front label (M = 4.81, SD = 0.894) than a Healthy Choice label (M = 4.51, SD = 0.905; F (1, 292) = 3.588, *p* < 0.01). Conversely, when the spokesperson type was a typical consumer, participants’ purchase intention of soda biscuits with a Healthy Choice label (M = 5.11, SD = 0.799) was higher than that with a Facts Up Front label (M = 4.07, SD = 1.168; F (1, 292) = 44.254, *p* < 0.01).

**Figure 4:**
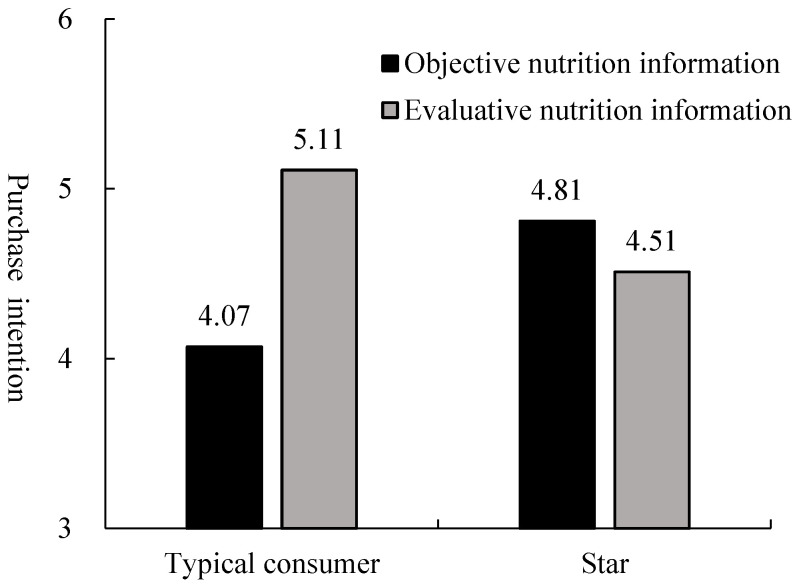
The moderating role of spokesperson type in the effect of nutrition label on purchase intention.

In other words, when a star was the spokesperson, consumers preferred healthy food with objective nutrition labels over evaluative nutrition labels. When a typical consumer was the spokesperson, consumers were more willing to buy healthy food with evaluative nutrition labels compared with objective nutrition labels (see Figure 4).

In addition, we conducted a 2 × 2 ANOVA on willingness to pay with the FOP nutrition label type (evaluative vs. objective) and spokesperson type (star vs. typical consumer) as the 2 factors. The results revealed no main effect of spokesperson type (F (1, 292) = 0.341, *p* = 0.560). The main effect of the nutrition label type (F (1, 292) = 5.255, *p* < 0.05) and the interaction effect were significant (F (1, 292) = 27.379, *p* < 0.01). A further simple effect analysis illustrated that participants in the star spokesperson condition were willing to pay more for soda biscuits with a Facts Up Front label (M = 18.04, SD = 4.382) than a Healthy Choice label (M = 16.56, SD = 4.035; F (1, 292) = 4.322, *p* < 0.05). Conversely, when the spokesperson type was a typical consumer, participants’ willingness to pay for soda biscuits with a Healthy Choice label (M = 19.49, SD = 3.984) was higher than that with a Facts Up Front label (M = 15.70, SD = 4.842; F (1, 292) = 28.315, *p* < 0.01).

**Figure 5:**
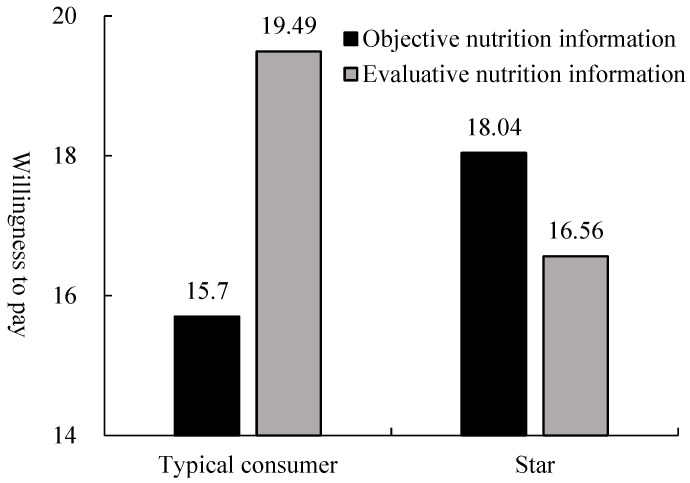
The moderating role of spokesperson type in the effect of nutrition label on willingness to pay.

That is to say, when a star was the spokesperson, consumers preferred to pay more for healthy food with objective nutrition labels than evaluative nutrition labels. When a typical consumer was the spokesperson, consumers were more willing to pay more for healthy food with evaluative nutrition labels compared with objective nutrition labels. Thus, H2, H2a, and H2b were supported (see Figure 5).

#### 3.3.3. Discussion

Firstly, study 3 found again that the provision of evaluative nutrition labels can contribute to higher purchase intention and more willingness to pay for healthy food, which means our main effect is very stable.

Secondly, this study confirmed the moderation effect of spokesman type on the relationship between the FOP nutrition label type and consumer purchase behavior of healthy food. More importantly, the influence of the FOP nutrition label type on consumers’ healthy food purchasing behavior is completely opposite in the case of different spokesperson types, which means there are competing mechanisms in the main effect.

## 4. Conclusions

Three experiments were conducted to investigate the influence of the FOP nutrition label type on consumers’ healthy food purchase behavior and the moderating effect of spokesperson type. Study 1 shows that consumers consider evaluative nutrition labels easier to understand but less informative than objective nutrition labels. Study 2 suggests that by providing evaluative nutrition labels (vs. objective nutrition labels) manufacturers can better promote consumers’ healthy food purchase behavior. Study 3 further demonstrates the moderating effect of spokesperson type. Specifically, when the spokesperson type is a typical consumer, consumers are more willing to buy healthy foods with evaluative nutrition labels than with objective nutrition label. When the spokesperson type is a star, objective nutrition labels (vs. evaluative nutrition labels) are more effective in promoting consumers’ healthy food purchase behavior.

We have made several theoretical contributions to the literature. Firstly, we explored the distinction between the evaluative nutrition label and objective nutrition label provided by manufacturers from the perspectives of comprehensibility and richness of information, thus theoretically enriching previous studies on FOP nutrition labels. Our findings suggest that an objective nutrition label is less understandable but more informative than an evaluative nutrition label. The discrepancy between these two types of FOP nutrition labels leads to consumers’ different purchase behaviors. Hence, our study advances the existing understanding of consumer preference for evaluative nutrition labels (vs. objective nutrition labels) by determining their differences in comprehensibility and information richness. Second, we further proposed how spokesperson type affects consumer preference for evaluative nutrition labels (vs. objective nutrition labels). Spokesperson type will lead to a change in the influence of the FOP nutrition label type on consumers’ healthy food purchase behavior by affecting the degree of consumers’ attention to the competence and warmth of manufacturers.

Our findings also have some important implications for marketing managers. Firstly, manufacturers should try to provide consumers with evaluative nutrition labels to promote consumers’ healthy food purchase behavior. For example, marketers can post evaluative nutrition labels to help consumers better understand the nutrition information of healthy food. In addition, manufacturers can also choose appropriate FOP nutrition labels according to different spokesperson types to maximize the marketing effect. For instance, when the spokesperson type is a star, manufacturers can give prominence to objective nutrition labels with richer information. However, when the spokesperson type is a typical consumer, manufacturers should emphasize evaluative nutrition labels to improve consumers’ healthy food purchase intention.

This study has the following three main research limitations. Firstly, we only used soda biscuits as the carrier of FOP nutrition labels. However, there are many kinds of healthy food, and future research can choose to study other healthy food as the carrier of the nutrition labels. Secondly, we only selected Facts Up Front and Healthy Choice labels to represent objective and evaluative nutrition labels, respectively. However, both FOP nutrition labels include numerous specific labels, and we did not discuss them one by one. Therefore, future research can consider choosing other evaluative nutrition labels (e.g., Health Star Rating) and objective nutrition labels (e.g., Guideline Daily Amount) to test our research conclusions. In addition, we only recruited participants from the online Credamo platform and did not use real marketing scenarios to further verify the research hypotheses. Therefore, future research can consider sampling participants offline for experiments or using real marketing data to verify our hypotheses, thereby enhancing the external validity of the research conclusions.

## Data Availability

The data that support the findings of this study are available from the corresponding authors upon reasonable request.

## References

[1] World Health Organization Obesity and Overweight Key Facts. https://www.who.int/news-room/factsheets/detail/obesity-and-overweight.

[2] Ungar R. Obesity Now Costs Americans More in Healthcare Spending than Smoking. https://www.forbes.com/sites/rickungar/2012/04/30/obesity-now-costs-americans-more-in-healthcare-costs-than-smoking/#5cfc435653d7.

[3] Popkin B.M., Kim S., Rusev E.R., Du S., Zizza C. (2006). Measuring the Full Economic Costs of Diet, Physical Activity and Obesity-Related Chronic Diseases. Obes. Rev..

[4] Andrews J.C., Burton S., Cook L.A. (2017). Nutrition Labeling in the United States and the Role of Consumer Processing, Message Structure, and Moderating Conditions.

[5] Nikolova H.D., Inman J.J. (2015). Healthy Choice: The Effect of Simplified Point-of-Sale Nutritional Information on Consumer Food Choice Behavior. J. Mark. Res..

[6] Bartels M., Tillack K., Jordan Lin C. (2018). Communicating Nutrition Information at the Point of Purchase: An Eye-tracking Study of Shoppers at Two Grocery Stores in the United States. Int. J. Consum. Stud..

[7] Ni Mhurchu C., Volkova E., Jiang Y., Eyles H., Michie J., Neal B., Blakely T., Swinburn B., Rayner M. (2017). Effects of Interpretive Nutrition Labels on Consumer Food Purchases: The Starlight Randomized Controlled Trial. Am. J. Clin. Nutr..

[8] Newman C.L., Howlett E., Burton S. (2016). Effects of Objective and Evaluative Front-of-Package Cues on Food Evaluation and Choice: The Moderating Influence of Comparative and Noncomparative Processing Contexts. J. Consum. Res..

[9] Feunekes G.I.J., Gortemaker I.A., Willems A.A., Lion R., van den Kommer M. (2008). Front-of-Pack Nutrition Labelling: Testing Effectiveness of Different Nutrition Labelling Formats Front-of-Pack in Four European Countries. Appetite.

[10] Bialkova S., Grunert K.G., Juhl H.J., Wasowicz-Kirylo G., Stysko-Kunkowska M., van Trijp H.C.M. (2014). Attention Mediates the Effect of Nutrition Label Information on Consumers’ Choice. Evidence from a Choice Experiment Involving Eye-Tracking. Appetite.

[11] Brown H.M., de Vlieger N., Collins C., Bucher T. (2017). The Influence of Front-of-Pack Nutrition Information on Consumers’ Portion Size Perceptions. Health Promot. J. Aust..

[12] Arrúa A., Curutchet M.R., Rey N., Barreto P., Golovchenko N., Sellanes A., Velazco G., Winokur M., Giménez A., Ares G. (2017). Impact of Front-of-Pack Nutrition Information and Label Design on Children’s Choice of Two Snack Foods: Comparison of Warnings and the Traffic-Light System. Appetite.

[13] Khandpur N., Sato P.D.M., Mais L.A., Martins A.P.B., Spinillo C.G., Garcia M.T., Rojas C.F.U., Jaime P.C. (2018). Are Front-of-Package Warning Labels More Effective at Communicating Nutrition Information than Traffic-Light Labels? A Randomized Controlled Experiment in a Brazilian Sample. Nutrients.

[14] Bialkova S., Grunert K.G., van Trijp H. (2013). Standing Out in the Crowd: The Effect of Information Clutter on Consumer Attention for Front-of-Pack Nutrition Labels. Food Policy.

[15] Lim J.H., Rishika R., Janakiraman R., Kannan P.K. (2020). Competitive Effects of Front-of-Package Nutrition Labeling Adoption on Nutritional Quality: Evidence from Facts Up Front–Style Labels. J. Mark..

[16] Neal B., Crino M., Dunford E., Gao A., Greenland R., Li N., Ngai J., Ni Mhurchu C., Pettigrew S., Sacks G. (2017). Effects of Different Types of Front-of-Pack Labelling Information on the Healthiness of Food Purchases—A Randomised Controlled Trial. Nutrients.

[17] Hassan L.M., Shium E.M.K., Michaelidou N. (2010). The Influence of Nutrition Information on Choice: The Roles of Temptation, Conflict and Self-Control. J. Consum. Aff..

[18] Mauri C., Grazzini L., Ulqinaku A., Poletti E. (2021). The Effect of Front-of-Package Nutrition Labels on The Choice of Low Sugar Products. Psychol. Mark..

[19] Dixon H., Scully M., Morley B., Wakefield M. (2021). Can Point-of-Sale Nutrition Information Encourage Reduced Preference for Sugary Drinks Among Adolescents?. Public Health Nutr..

[20] Ducrot P., Julia C., Méjean C., Kesse-Guyot E., Touvier M., Fezeu L.K., Hercberg S., Péneau S. (2016). Impact of Different Front-of-Pack Nutrition Labels on Consumer Purchasing Intentions: A Randomized Controlled Trial. Am. J. Prev. Med..

[21] Hwang J. (2013). The Effects of Nutrient Ad Disclosures of Fast Food Menu Items on Consumer Selection Behaviors Regarding Subjective Nutrition Knowledge and Body Mass Index. Br. Food J..

[22] Xie G.-X., Kronrod A. (2012). Is the Devil in the Details?. J. Advert..

[23] Huang L., Tan C.-H., Ke W., Wei K.-K. (2014). Do we order product review information display? How?. Inf. Manag..

[24] Pang J., Hammond D. (2013). Efficacy and Consumer Preferences for Different Approaches to Calorie Labeling on Menus. J. Nutr. Educ. Behav..

[25] Berning J.P., Chouinard H.H., McCluskey J.J. (2008). Consumer Preferences for Detailed Versus Summary Formats of Nutrition Information on Grocery Store Shelf Labels. J. Agric. Food. Ind. Organ..

[26] Andrews J.C., Burton S., Kees J. (2011). Is Simpler Always Better? Consumer Evaluations of Front-of-Package Nutrition Symbols. J. Public Policy Mark..

[27] Prabhaker P.R., Sauer P. (1994). Hierarchical Heuristics in Evaluation of Competitive Brands Based on Multiple Cues. Psychol. Mark..

[28] van Herpen E., van Trijp H.C.M. (2011). Front-of-Pack Nutrition Labels. Their Effect on Attention and Choices When Consumers Have Varying Goals and Time Constraints. Appetite.

[29] Friestad M., Wright P. (1994). The Persuasion Knowledge Model: How People Cope with Persuasion Attempts. J. Consum. Res..

[30] Cheema A., Patrick V.M. (2008). Anytime Versus Only: Mind-Sets Moderate the Effect of Expansive Versus Restrictive Frames on Promotion Evaluation. J. Mark. Res..

[31] Wei J.M.Y. (2003). Codeswitching in Campaigning Discourse: The Case of Taiwanese President Chen Shuibian. Lang. Linguist..

[32] Rosenberg M. (1965). 2. The measurement of self-esteem. Society and the Adolescent Self-Image.

[33] Schnaubert L., Krukowski S., Bodemer D. (2021). Assumptions and Confidence of Others: The Impact of Socio-Cognitive Information on Metacognitive Self-Regulation. Metacogn. Learn..

[34] Anderson C., Brion S., Moore D.A., Kennedy J.A. (2012). A Status-Enhancement Account of Overconfidence. J. Pers. Soc. Psychol..

[35] Anderson C., Kilduff G.J. (2009). Why Do Dominant Personalities Attain Influence in Face-to-Face Groups? The Competence-Signaling Effects of Trait Dominance. J. Pers. Soc. Psychol..

[36] Newman C.L., Howlett E., Burton S. (2014). Shopper Response to Front-of-Package Nutrition Labeling Programs: Potential Consumer and Retail Store Benefits. J. Retail..

[37] Berry C., Burton S., Howlett E. (2018). The Effects of Voluntary Versus Mandatory Menu Calorie Labeling on Consumers’ Retailer-Related Responses. J. Retail..

[38] Aaker J.L., Garbinsky E.N., Vohs K.D. (2012). Cultivating Admiration in Brands: Warmth, Competence, and Landing in the “Golden Quadrant”. J. Consum. Psychol..

[39] Kervyn N., Fiske S.T., Malone C. (2012). Brands as Intentional Agents Framework: How Perceived Intentions and Ability Can Map Brand Perception. J. Consum. Psychol..

[40] Aaker J., Vohs K.D., Mogilner C. (2010). Nonprofits Are Seen as Warm and For-Profits as Competent: Firm Stereotypes Matter. J. Consum. Res..

[41] Ybarra O., Chan E., Hyekyung P., Burnstéin E., Monin B., Stańik C. (2008). Life’s Recurring Challenges and the Fundamental Dimensions: An Integration and Its Implications for Cultural Differences and Similarities. Eur. J. Soc. Psychol..

[42] Abele A.E., Bruckmüller S. (2011). The Bigger One of the ‘Big Two’? Preferential Processing of Communal Information. J. Exp. Soc. Psychol..

[43] Friedman H.H., Friedman L. (1979). Endorser Effectiveness by Product Type. J. Advert. Res..

[44] Wilson E.J., Sherrell D.L. (1993). Source Effects in Communication and Persuasion Research: A Meta-Analysis of Effect Size. J. Acad. Mark. Sci..

[45] Ahn J., Kim J., Sung Y. (2021). AI-Powered Recommendations: The Roles of Perceived Similarity and Psychological Distance on Persuasion. Int. J. Advert..

[46] Ayeh J.K. (2015). Travellers’ Acceptance of Consumer-Generated Media: An Integrated Model of Technology Acceptance and Source Credibility Theories. Comput. Hum. Behav..

[47] Schouten A.P., Janssen L., Verspaget M. (2020). Celebrity Vs. Influencer Endorsements in Advertising: The Role of Identification, Credibility, and Product-Endorser Fit. Int. J. Advert..

[48] Jiang J., Huang Y.-H., Wu F., Choy H.-Y., Lin D. (2015). At the Crossroads of Inclusion and Distance: Organizational Crisis Communication during Celebrity-Endorsement Crises in China. Public Relat. Rev..

[49] Kim H.J., Dempsey M.A. (2019). Processing Difficulty Increases Perceived Competence of Brand Acronyms. Can. J. Adm. Sci..

[50] Freddi S., Tessier M., Lacrampe R., Dru V. (2014). Affective Judgement about Information Relating to Competence and Warmth: An Embodied Perspective. Br. J. Soc. Psychol..

[51] Chen J., Teng L., Liao Y. (2018). Counterfeit Luxuries: Does Moral Reasoning Strategy Influence Consumers’ Pursuit of Counterfeits?. J. Bus. Ethics.

[52] Netemeyer R.G., Krishnan B., Pullig C., Wang G., Yagci M., Dean D., Ricks J., Wirth F. (2004). Developing and Validating Measures of Facets of Customer-Based Brand Equity. J. Bus. Res..

